# MEMS Tunable Diffraction Grating for Spaceborne Imaging Spectroscopic Applications

**DOI:** 10.3390/s17102372

**Published:** 2017-10-17

**Authors:** Sanathanan S. Muttikulangara, Maciej Baranski, Shakil Rehman, Liangxing Hu, Jianmin Miao

**Affiliations:** 1 School of Mechanical and Aerospace Engineering, Nanyang Technological University, 50 Nanyang Avenue, Singapore 639798, Singapore; mbaranski@ntu.edu.sg (M.B.); lhu001@e.ntu.edu.sg (L.H.); jmiao@pmail.ntu.edu.sg (J.M.); 2 Singapore-MIT Alliance for Research and Technology (SMART), 1 CREATE Way, Singapore 138602, Singapore; shakil1512@gmail.com

**Keywords:** tunable diffraction grating, optical components, microelectromechanical devices

## Abstract

Diffraction gratings are among the most commonly used optical elements in applications ranging from spectroscopy and metrology to lasers. Numerous methods have been adopted for the fabrication of gratings, including microelectromechanical system (MEMS) fabrication which is by now mature and presents opportunities for tunable gratings through inclusion of an actuation mechanism. We have designed, modeled, fabricated and tested a silicon based pitch tunable diffraction grating (PTG) with relatively large resolving power that could be deployed in a spaceborne imaging spectrometer, for example in a picosatellite. We have carried out a detailed analytical modeling of PTG, based on a mass spring system. The device has an effective fill factor of 52% and resolving power of 84. Tuning provided by electrostatic actuation results in a displacement of 2.7 μm at 40 V. Further, we have carried out vibration testing of the fabricated structure to evaluate its feasibility for spaceborne instruments.

## 1. Introduction

In recent years, extensive research has been carried out in the industry for the development and launching of small satellites into space. Small satellites have been found to be very successful for dedicated applications such as remote sensing due to features like low mass (1kg to 50kg), small size, low power consumption and low manufacturing costs [1]. One such effort aims to build a dedicated small-satellite-compatible, miniaturized spectroscopic cameras for remote sensing [2]. The integration of MEMS technology is promising for the future development of such systems and for small satellite programs in general [3,4].

One candidate device for a satellite-carried spectrometer is a tunable diffraction grating [5]. More generally, tunable gratings are attractive for sensing, displays [6] and tunable lasers [7,8,9]. Since a small footprint is essential for the tunable grating in the micro-satellite setting, the use of MEMS technology for the fabrication of our grating is particularly appealing. Earlier approaches to tuning gratings have been by means of grating light valve [10], microfluidic actuation [11], piezoelectric actuation [12], electrostatic actuation [13,14,15], thermal actuation [16], and elastomeric actuation [17]. In our work we have chosen electrostatic actuation that can work in wide range of temperatures and pressures with low power, and can provide sufficient actuation range for our considered application.

The most common electrostatic actuators are limited by small displacement to a few micrometers. This is because electrostatic comb-drive based actuator encounters the pull-in instability if sufficiently high actuation voltages are attempted [18]. This limits the maximum size of a electrostatic pitch tunable diffraction grating to about a few millimeters. However, they can be used for integrated computational imaging spectroscopic applications by making use of optical diversity techniques [19]. Diverse measurements with different optical transfer functions can be obtained by varying the pitch of the diffraction grating in these optical systems. By this method and with computational algorithms, multiple undersampled images can be used to obtain a super-resolution image [20]. For such imaging spectroscopic applications, large resolving power diffraction gratings with high fill factors are found to be advantageous. To achieve this, careful analysis and design of the micromechanical structure is necessary.

This paper describes the analysis of MEMS based analog pitch tunable diffraction grating using Silicon-On-Insulator (SOI) technology. The following sections describe the design, modeling, fabrication and testing of our device. Further, the feasibility of using silicon micromachined PTG for space-borne instruments is investigated by subjecting it to mechanical vibrations.

## 2. Pitch Tunable Diffraction Grating (PTG)

**Figure 1:**
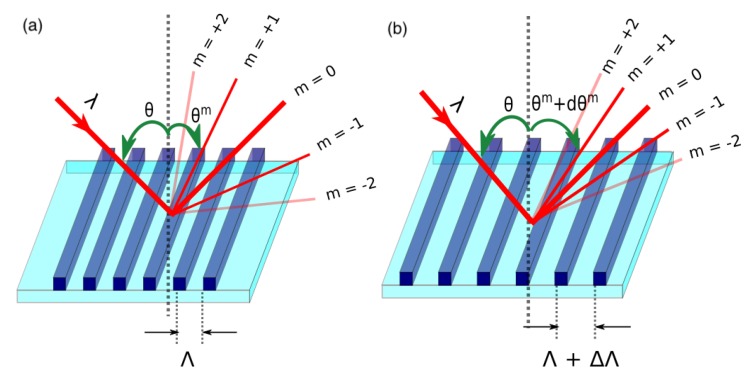
Schematic representation of the basic working principle of an analog pitch tunable diffraction grating: (**a**) before tuning, (**b**) after tuning.

Light incident on the diffraction grating is dispersed according to the diffraction relation given by

(1) $$\sin\theta^{m}+\sin\theta =\frac{m\lambda }{\Lambda }$$

where θ is the incident angle, $\theta^{m}$ is the diffracted angle of order *m*, λ is the incident wavelength, and Λ is the pitch of the diffraction grating. The value of diffraction orders (*m*) are integers and *m* = 0 gives the non-dispersive term. The schematic working principle of a PTG is depicted in Figure 1.

Tuning can be incorporated by elongating or compressing the pitch, thereby changing the diffraction angle ($\theta^{m}$). The pitch change of the diffraction grating from Λ to Λ+dΛ leads to a change in diffraction angle $d\theta^{m}$, which is given by

(2) $$d\theta^{m}=\frac{m\lambda }{\Lambda^{2}}d\Lambda$$

where $d\Lambda =\frac{x}{N}$ is the pitch change, *x* is the displacement achieved in actuation and *N* is the number of grating lines. It is evident that larger deflection provides better tuning range. However, larger deflection also leads to change in the duty cycle (*DC*), and a significant drop in diffraction efficiency (η) [21] expressed as

(3) $$\eta ={\left(\frac{\sin(2m\pi \;DC)}{2m\pi }\right)}^{2}+{\left(\frac{1-\cos(2m\pi \;DC)}{2m\pi }\right)}^{2}$$

The DC in this context is defined as the ratio of beam width to the pitch. Maximum efficiency of about 10% is obtained when duty cycle is 50%.

### 2.1. Micromechanical Implementation

**Figure 2:**
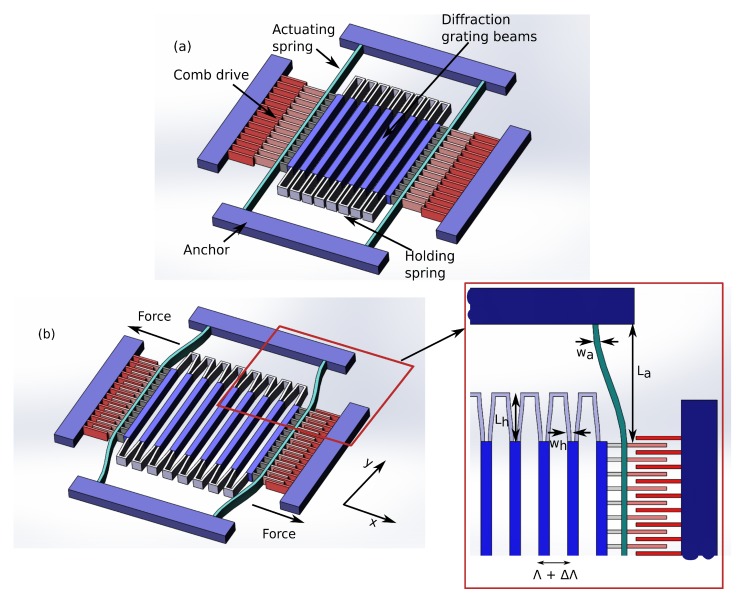
Schematic of tunable diffraction grating with electrostatic actuation: (**a**) before actuation; (**b**) after actuation.

In our design, we employ in-plane electrostatic comb-drive pair actuation mechanism for its simplicity and relative ease of fabrication [22,23,24]. The schematic of the electrostatic actuation based PTG is depicted in Figure 2. The grating grooves are implemented as a set of beams, which are supported by holding springs. The grating beams together with the holding springs are suspended by the actuating springs that are connected to the anchors. The actuation springs are equipped with a comb-drive-fingers structure that generates electrostatic force with the application of voltage.

The electrostatic force generated by the comb-drive pairs can be modeled by a planar parallel plate capacitor [25]. This generated force is balanced by mechanical force, thereby effectively increasing the overlap area. The net electrostatic force generated by the comb-drive pairs consists of a combination of pull-in force by the parallel plates, fringing fields (edge effects) and ground-plane levitation force (between the suspended structure and the substrate). Neglecting the fringing fields and ground-plane levitation force, the capacitive force generated between the comb-drive pairs is given by

(4) $$F_{e}=\frac{n\epsilon_{0}t}{g}V^{2}=F_{m}=k_{eff}x,$$

where $F_{e}$ is the electrostatic force, *n* is the number of comb fingers, $\epsilon_{0}$ is the permittivity of free space, *t* is the thickness of the device, *g* is the separation between the parallel plates, *V* is the applied voltage, $F_{m}$ is the mechanical force generated, $k_{eff}$ is the effective spring constant of the structure, and *x* is the displacement obtained. This force, in turn, pulls the actuator spring in the *x* direction to balance the electrostatic force. The larger tuning range can be achieved by increasing the actuation voltage.

### 2.2. Optical Design

The intensity profile of a diffraction grating when *N* slits are illuminated by a collimated beam with normal incidence, can be obtained by

(5) $$I=I_{0}\frac{\sin^{2}\left(\frac{N\pi \Lambda }{\lambda }\sin\theta^{m}\right)}{\sin^{2}\left(\frac{\pi \Lambda }{\lambda }\sin\theta^{m}\right)}\frac{\sin^{2}\left(\frac{\pi \Lambda }{2\lambda }\sin\theta^{m}\right)}{{\left(\frac{\pi \Lambda }{2\lambda }\sin\theta^{m}\right)}^{2}}$$

An important parameter that needs to be considered while designing the diffraction grating is the resolving power (*R*), defined as number of grating lines (*N*). The resolving power of the diffraction grating for two wavelengths ($\lambda_{1}$,$\lambda_{2}$) which are closely spaced is given by

(6) $$R=\frac{1}{2}\frac{\lambda_{1}+\lambda_{2}}{\lambda_{1}-\lambda_{2}}=mN$$

**Figure 3:**
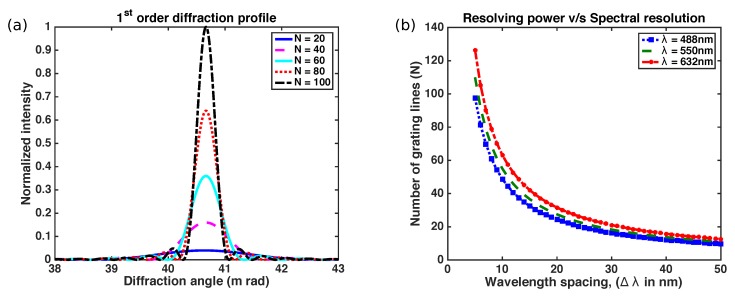
(**a**) First order diffraction profile in variation with the number of grating lines illuminated; (**b**) Plot depicting minimum number of grating lines for the closely separated wavelength at different wavelength regimes.

The full width half maximum (FWHM) of the diffraction profile depends on *N*. Figure 3a shows the first order diffraction profile when a wavelength of 488 nm is incident on *N* slits. It is evident from the plot that FWHM reduces i.e., resolution increases with the increase in *N*. Resolution also depends on the wavelength regime which is depicted in Figure 3b, where the number of grating lines plotted against resolution for three wavelengths. Here the center wavelength and wavelength spacing are taken as $(\lambda_{1}+\lambda_{2})/2$ and $\left(\lambda_{1}-\lambda_{2}\right)$ respectively.

Further, it is evident that to obtain a spectral resolution less than 10 nm for 632 nm wavelength in 1st order diffraction, there needs to be a minimum 64 grating lines.

### 2.3. Micromechanical Design

The stiffness constant of the holding flexure can be derived by treating the flexure as a four guided cantilever structure. The expression for computing the stiffness value for a single holding spring with thickness *t*, is obtained from

(7) $$k_{h}^{x}=12\frac{EI_{h}^{x}}{L_{h}^{3}},I_{h}^{x}=\frac{1}{12}tw_{h}^{3}$$

where $k_{h}^{x}$ is the holding spring stiffness in *x* direction, *E* is Young’s modulus, $I_{h}^{x}$ is the moment of inertia and $w_{h}$($L_{h}$) is the width (length) of the holding springs. The spring constant of the actuating spring can be treated as a two guided beam structure connected in parallel. Accordingly, the spring stiffness value of the actuating arm in the lateral direction ($k_{a}^{x}$) for one-side is

(8) $$k_{a}^{x}=12\frac{EI_{a}^{x}}{L_{a}^{3}},I_{a}^{x}=\frac{1}{12}tw_{a}^{3}$$

where $k_{a}^{x}$ is the actuating spring stiffness in *x* direction, $I_{a}^{x}$ is the moment of inertia of the actuating spring and $w_{a}$($L_{a}$) is the width (length) of the actuating springs. The spring stiffness of the actuating arm in the *y* direction is computed as

(9) $$k_{a}^{y}=\frac{Ew_{a}t}{L_{a}}$$

where $k_{a}^{y}$ is the actuating spring stiffness in *y* direction. The springs of the actuating arm are designed to provide maximum tangential displacement (minimum tangential stiffness, $k_{a}^{x}$) and minimum normal displacement (maximum normal stiffness, $k_{a}^{y}$). The stiffness ratio has to be large enough to avoid lateral pull-in instability which limits the actuation range of the device. It is obtained as

(10) $$\frac{k_{a}^{y}}{k_{a}^{x}}={\left(\frac{L_{a}}{w_{a}}\right)}^{2}$$

For design, the effective spring constant ($k_{eff}$) has to be computed first, followed by the evaluation of the other parameters. Yu et al. reported that the displacement of the comb drive fingers drops significantly when the grating beams are connected with the actuating spring [26]. This discrepancy can be explained by a detailed analytical expression of the effective spring constant. The effective stiffness of the structure can be derived from the mass-spring model which solely depends on actuating spring stiffness ($k_{a}$), holding spring stiffness ($k_{h}$), and the number of grating periods (*N*).

#### 2.3.1. Mass-Spring Model

**Figure 4:**
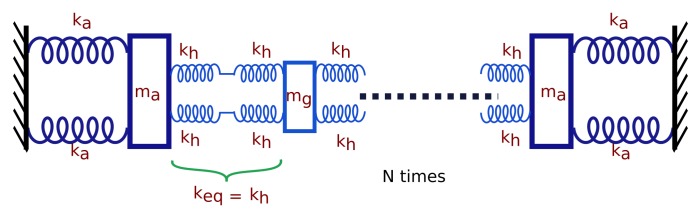
Schematic representation of equivalent mass-spring model of tunable diffraction grating structure.

To compute the unknown effective spring constant ($k_{eff}$ in Equation (4)), the structure in Figure 2 can be modeled as a mass-spring system portrayed in Figure 4.

The equivalent spring constant between two grating beams ($k_{eq}$) is the parallel combination of springs connected in series. The stiffness of a spring connected to *N* beams can be computed as a combination of *N* springs of individual stiffness $k_{eq}$ ($=k_{h}$) connected in series. The force generated from the comb drives on either side of the device pulls the first $\frac{N}{2}$ grating beams in one direction and the remaining beams in the other direction to obtain maximum tuning range. The effective spring constant of the structure when electrostatic force is applied in both directions can be computed as

(11) $$k_{eff}=2k_{a}+\left(\frac{2}{N}\right)k_{h}$$

After obtaining $k_{eff}$, the remaining parameters ($L_{h},L_{a},w_{h},w_{a}$) are chosen based on the fabrication constraints. The length of the grating beams is chosen to have an effective diffraction area of 1 mm× 1 mm. The minimum feature size of the structure is selected as 2 μm, which is the critical dimension for photolithography. The design values are summarized in Table 1.

#### 2.3.2. In-Plane Modal Analysis

The in-plane mechanical resonant frequencies of the PTG are computed using a mass-spring model for stability analysis. The natural frequencies of the mass-spring system can be derived by equations of motion for an undamped linear system using

(12) $$M\frac{d^{2}x}{dt^{2}}+Kx=0,$$

where *x* is the time dependent vector that describes the motion, **M** is the mass matrix and **K** is the stiffness matrix given by

$$\begin{array}{c} M=\left[\begin{array}{cccccc} m_{a} & 0 & 0 & \cdots & 0 & 0\\ 0 & m_{g} & 0 & \cdots & 0 & 0\\ 0 & 0 & m_{g} & \cdots & 0 & 0\\ \vdots & \vdots & \vdots & \ddots & \vdots & \vdots \\ 0 & 0 & 0 & \cdots & m_{g} & 0\\ 0 & 0 & 0 & \cdots & 0 & m_{a} \end{array}\right]\\ K=\left[\begin{array}{cccccc} 2k_{a}+k_{h} & -k_{h} & 0 & \cdots & 0 & 0\\ -k_{h} & 2k_{h} & -k_{h} & \cdots & 0 & 0\\ 0 & -k_{h} & 2k_{h} & \cdots & 0 & 0\\ \vdots & \vdots & \vdots & \ddots & \vdots & \vdots \\ 0 & 0 & 0 & \cdots & 2k_{h} & -k_{h}\\ 0 & 0 & 0 & \cdots & -k_{h} & k_{h}+2k_{a} \end{array}\right] \end{array}$$

where $m_{a}$ is the sum of mass of the actuating arm and the mass of comb fingers attached to it, $m_{g}$ is the sum of the mass of grating beam and the four supporting holding beams, $k_{a}$ (=$k_{a}^{x}$) is the actuating spring stiffness along *x* direction, and $k_{h}$ (=$k_{h}^{x}$) is the holding spring stiffness along *x* direction. To find the harmonic solution, we assume that *x* takes the from Xsin(ωt) which results in

(13) $$-MX\omega^{2}\sin\omega t+KX\sin\omega t=0$$

On further simplification, the above equation gives KX = $\omega^{2}MX$. The generalized eigenvectors are given by substituting vector *v* and λ in the above equation gives the form **K***v* = λ**M***v*. The eigenvalue solution λ is computed by MATLAB for **M** and **K**. The natural frequency of the system for the *i*th eigenvalue is then

(14) $$f_{i}=\frac{1}{2\pi }\sqrt{\lambda_{i}}$$

The analytical model for structural analysis and resonant frequency was validated by 3D finite element simulations before proceeding to the fabrication.

## 3. Microfabrication

**Figure 5:**
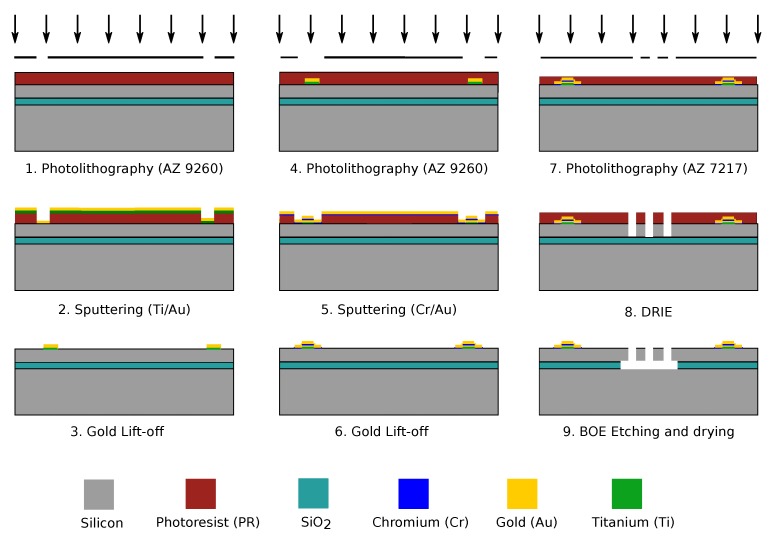
Schematic representation of the fabrication of tunable amplitude diffraction grating.

The fabrication of the device was performed using surface machining technology on a 100 mm diameter SOI wafer with 10 μm device layer thickness, 2 μm buried oxide, 450 μm handle layer, <100> crystal orientation, boron doping and a resistivity between 1Ωcm to 20Ωcm. The wafer was initially dipped in buffered hydrofluoric acid (HF) solution for 2 min and was rinsed using de-ionised (DI) water. The wafer was spin dried and then placed on a hot plate at 110 ${}^{\circ }C$ for 30 min to make the surface dehydrated. Before performing the spin coating, the wafer was treated with HMDS primer for 120 s to make the surface hydrophilic, thus improving the adhesion of photoresist with the wafer. A brief schematic representation of fabrication process is depicted in Figure 5.

In the first step, contact pads for external electrical connection are patterned by photolithography using AZ9260 photoresist on the SOI wafer and then sputtered with Titanium (Ti)/ Gold (Au), further followed by lift-off (steps 1–3 in Figure 5). Ti serves as the adhesion layer between Si and Au. To protect the Ti from buffered oxide etchant (BOE) in the later step of fabrication, the wafer is again patterned using AZ9260 photoresist and sputtered with Chromium (Cr)/ Gold (Au) on top of the existing pads, thereby, completely covering Ti layer (steps 4–6 in Figure 5). Further, photolithography for the PTG structure is carried out with a thinner photoresist, AZ7217 followed by Deep Reactive Ion Etching (DRIE) process to obtain nearly vertical walls. Finally, the sacrificial oxide layer beneath the structure is removed by BOE solution (steps 7–9 in Figure 5).

The main challenge during drying is the stiction between adjacent grating beams and also in the holding springs due to the surface tension between water and the device. To overcome this problem, critical CO${}_{2}$ drying process is adopted. By this method, the wafer is dried with liquid CO${}_{2}$ under critical point temperature and pressure by which the physical properties of gaseous and liquid states remain unchanged.

**Figure 6:**
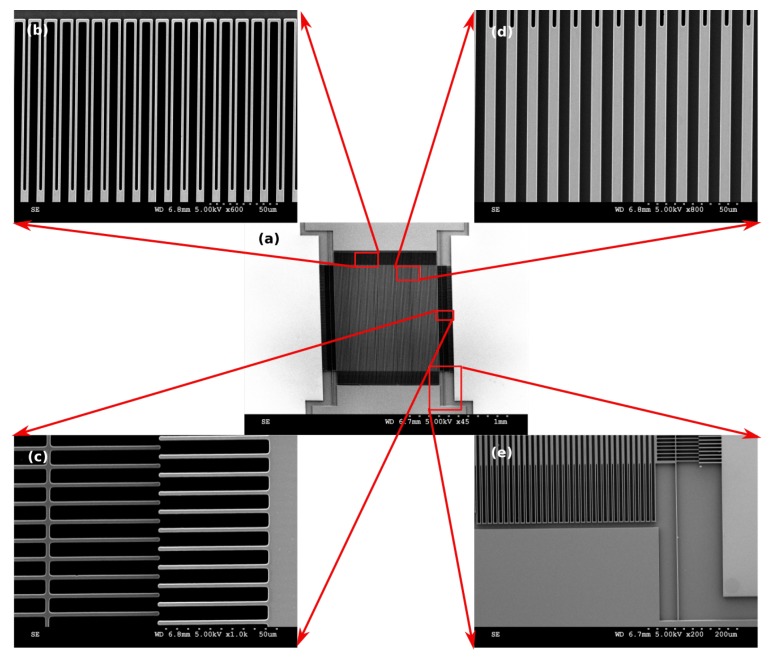
SEM images of the fabricated MEMS diffraction grating (**a**) device; (**b**) holding springs; (**c**) comb-drive fingers; (**d**) grating beams; and (**e**) actuation spring.

The SEM images of the fabricated device are depicted in Figure 6.

## 4. Device Characterization and Testing

We found that device performance was dependent on micro fabrication tolerances. With the conditions of the DRIE system we employed, etching produces a negative sloped profile in the beams where the trench space is large and a positive profile where trenches are closely spaced [27,28]. This discrepancy in etching profile might result in relatively large variation in the expected device performance [29]. The comparison between the designed values from the analytical modeling and the fabricated values are shown in Table 1. The modeling and performance analysis of such etching profiles has been carried out in [30]. Based on the fabricated structure, we modified the analytical modeling of displacement as

(15) $$x=\frac{F_{\left(new\right)}}{k_{eff(new)}}=\frac{n\epsilon_{0}V^{2}}{2Et\tan\theta }\frac{\log\left(1+2\frac{t}{g}\tan\theta \right)}{\frac{2}{L_{a}^{3}}{\left(w_{a}+t\tan\phi \right)}^{3}+\frac{2}{NL_{h}^{3}}{\left(w_{h}+t\tan\psi \right)}^{3}}$$

where θ, ϕ and ψ are the etching profile angles in comb-drive fingers, actuation springs and holding springs, respectively. The spring stiffness with the modified design is calculated to be 1.56 $Nm^{-1}$ against the initially designed value of 0.94 $Nm^{-1}$. Further, the negatively tapered etching profile in the comb-drives leads to reduction in electrostatic force from 8.8 μN to 4.6 μN for the driving voltage of 40 V.

### 4.1. Static Measurements

**Figure 7:**
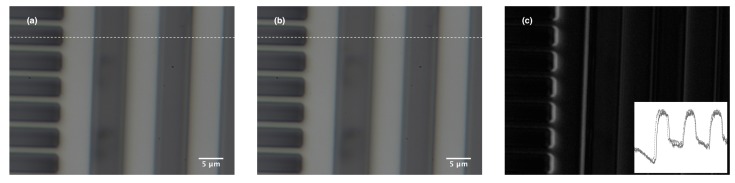
Images from the optical microscope of the grating beams (**a**) before the application of the voltage; (**b**) with the application of voltage; (**c**) difference between the two images (the inset shows a line plot along the dashed line in (**a**,**b**)).

The fabricated PTG was tested in a probe station by observing the deflection of the grating beams with the applied voltage. Figure 7a,b depict the tuning of PTG without and with the driving voltage along with the difference between two images. The inset in Figure 7c shows a line profile along the white dashed line from the two images of the grating.

**Figure 8:**
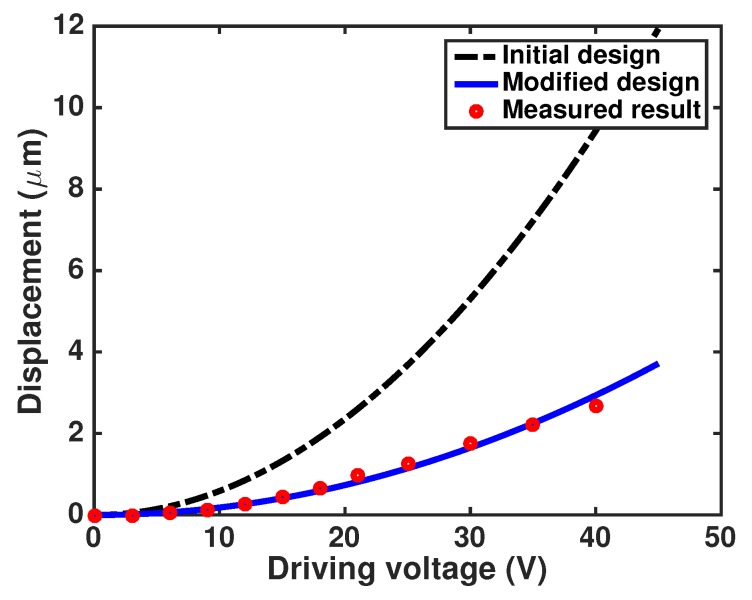
Displacement obtained in pitch tunable diffraction grating (PTG) plotted against voltage.

Stretching of the grating beams is achieved by the moving comb fingers during actuation. The displacement obtained in the comb fingers is plotted with respect to the voltage in Figure 8. The plot clearly shows that displacement follows a quadratic relation with the applied voltage and also the measured values match with the modified design.

For safe working of the device, the driving voltage has to be less than the pull-in instability voltage which is given by

(16) $$V_{PI}=\frac{g^{2}k_{a}^{x}}{2\epsilon_{0}w_{a}n}\left(\sqrt{2\frac{k_{a}^{y}}{k_{a}^{x}}+\frac{x_{0}^{2}}{g^{2}}}-\frac{x_{0}}{g}\right)$$

Since our design has higher stiffness ratio ($k_{a}^{y}/k_{a}^{x}$ in Equation (10)), and the operating voltage was far less than the pull-in voltage, the device did not face any lateral pull-in instability. However, we observed that the device was sticking to the handle layer of the wafer when higher driving voltages were applied. This can be rectified by modifying the fabrication steps by incorporating additional photolithography on the handle layer followed by etching.

### 4.2. Optical Characterization

**Figure 9:**
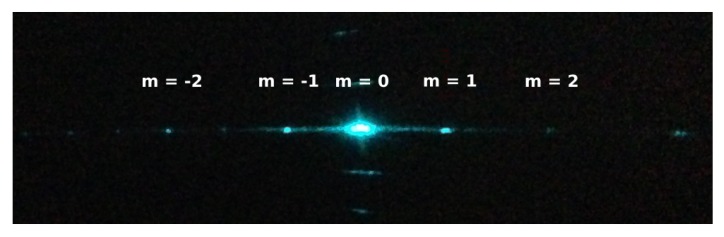
Diffraction pattern obtained with a 488 nm laser beam incident on the PTG.

The fabricated grating was also tested optically. We illuminated the PTG with a laser beam of wavelength 488 nm, and the diffraction pattern was observed on a dark screen that is shown in Figure 9.

To validate the shift in the diffracted angle, the 1st order diffraction spot (m = +1) was detected on a camera. Further, voltage was applied through the contact pads; and the resulting shift in the first order diffraction was measured to be four pixels. This corresponds to a change of 218 μrad in diffraction angle.

A comparison of our PTG with other research works is shown in Table 2.

### 4.3. Mechanical Vibration Test

The reliability tests that are generally carried in the satellite missions are radiation tests, vacuum tests, thermal cycling tests, thermal shock tests, mechanical shock tests and mechanical vibration tests. Out of these, the MEMS devices are more susceptible to mechanical shock and vibrations. The level of mechanical shock depends on the type of the mission. In general, for a satellite assignment there are three levels of shock that are experienced: launching, separation from the rocket, and landing. Typical shock values experienced by previously reported missions are 4.5 G for the Russian Soyuz vehicle [32] and 3 G for NASA Space Shuttle [33]. To measure the level of shock, a finite element simulation (ANSYS 17.2) was performed with different shock levels.

**Figure 10:**
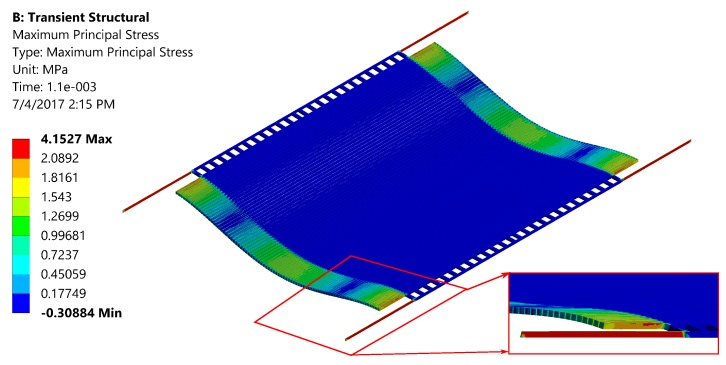
Stress distribution of the structure when subjected to a shock of 5 G.

The simulations were carried out with the same geometry and with the designed values described in Table 1. Anisotropic silicon was chosen as the material for all the simulations. In one simulation, a shock of 5 G was applied along the weakest out-of-plane direction of the PTG. It was found that maximum stress is concentrated on the actuating springs and there was no significant stress in the optical grating region (Figure 10). The result shows that maximum principal stress is 4.15 MPa, far less than the fracture strength of Silicon (7000 MPa). Since our fabricated device is stiffer than the design values, the device would most likely surpass the shock test.

**Figure 11:**
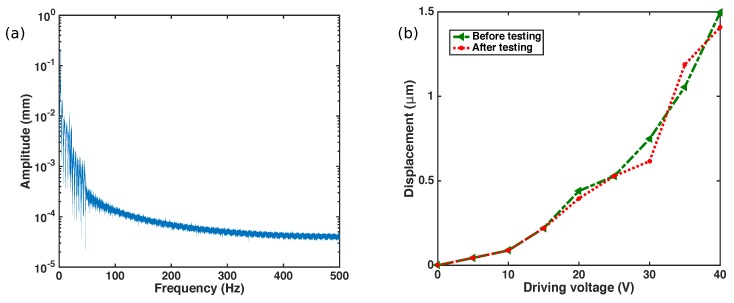
Vibration test analysis of the PTG with (**a**) frequency response of the vibrations measured using laser displacement sensor; (**b**) Performance of our device measured experimentally before and after the vibration test.

We also studied the mechanical strength of our device by subjecting it to mechanical vibrations. The mechanical vibration is an important concern during the satellite launch. The typical values of the frequency of vibration are 1 Hz to 100 Hz [34]. Hence, it is important to make sure that the natural frequency of the device does not fall within this range of frequencies leading to fracture of the device. To verify that, the PTG is not susceptible to vibrations, we carried out vibration test using a mini-shaker by varying its frequency using a function generator. The amplitude of excitation is controlled by increasing the voltage in the function generator. We also observed that the amplitude of excitation reduces with the increase in frequency. The vibration response of the mini shaker was measured by a laser displacement sensor and the corresponding amplitude-frequency plot is shown in Figure 11a.

The in-plane vibrations and out-of-plane vibrations were performed by placing the device in different orientations. A different device was used for vibration testing as we did not prefer damaging our best device. The process ran for 1 h and no mechanical failure of the structure was observed. After undergoing the vibration test, the device was evaluated in a probe station. Figure 11b shows the displacement-voltage relation of the device before and after the test. The test results show that the device was capable of sustaining mechanical vibrations.

## 5. Conclusions

We designed and fabricated a silicon-based pitch tunable diffraction grating using micromachining technology. A detailed analytical modeling was carried out for the PTG using a mass-spring model. The device was fabricated with a 10 μm thick SOI wafer. The micro-mechanical performance was analyzed for the fabricated structure with the modified design. The tuning produces a displacement of 2.7 μm for an actuation voltage of 40 V. With the application of different voltages, the displacement was found to follow the expected quadratic law, in good agreement with the nominal parameters. The diffraction grating was tested on an optical bench by applying actuation voltage to observe the positional shift in the diffraction orders. Finally, the device was subjected to vibration tests and was found to meet the criteria for spaceborne applications.

References

## Figures and Tables

**Table 1:** Comparison of designed and measured values of parameters of the microelectromechanical system (MEMS) pitch tunable diffraction grating.

**Device Parameters**	**Symbol**	**Designed** (μm)	**Measured** (μm)
grating beam length	$L_{g}$	1000	1000
grating beam width	$w_{g}$	6	5.9
grating pitch	Λ	12	12
device thickness	*t*	10	10
holding spring width	$w_{h}$	2	1.9
holding spring length	$L_{h}$	122	122
actuating spring width	$w_{a}$	2	2.7
actuating spring length	$L_{a}$	333	333
comb finger length	$L_{c}$	50	50
comb finger width	$w_{c}$	2	0.7
initial overlap distance	$x_{0}$	2	1.8
**Device Parameters**	**Symbol**	**Designed**	
No. of grating beams	N	84	
No. of comb fingers	n	125	

**Table 2:** Performance analysis of the PTG with other works.

Reference	Actuation Mechanism	Resolving Power	Fill Factor	Actuation Voltage	Displacement
Author (Year)		$\left(\frac{\lambda_{0}}{\delta \lambda }\right)$	(Effective Area/Total Area) (%)	(V)	(μm)
Wei et al. (2006) [13]	Electrostatic	17	20	10	0.46
Yu et al. (2010) [14]	Electrostatic	30	22	70	10.4
Yu et al. (2010) [26]	Electrostatic	29	≈10	100	21
Tormen et al. (2006) [15]	Electrostatic	33	≈30	100	2
Yang et al. (2008) [16]	Thermal	54	≈21	19	300
Wong et al. (2003) [31]	Piezoelectric	-	-	10	≈0.28
this work	Electrostatic	84	52	40	2.7

## References

[1] Kramer H.J., Cracknell A.P. (2008). An overview of small satellites in remote sensing*. Int. J. Remote Sens..

[2] Guelman M., Ortenberg F. (2009). Small satellite’s role in future hyperspectral Earth observation missions. Acta Astronaut..

[3] De Rooij N., Gautsch S., Briand D., Marxer C., Mileti G., Noell W., Shea H., Staufer U., Van Der Schoot B. MEMS for space. Proceedings of the TRANSDUCERS 2009–2009 International Solid-State Sensors, Actuators and Microsystems Conference.

[4] Shea H. (2009). MEMS for pico-to micro-satellites. SPIE MOEMS-MEMS: Micro- and Nanofabrication.

[5] Truxal S.C., Kurabayashi K., Tung Y.C. (2008). Design of a MEMS tunable polymer grating for single detector spectroscopy. Int. J. Optomechatron..

[6] Brazas J.C., Kowarz M.W. (2004). High-resolution laser-projection display system using a grating electromechanical system (GEMS). Micromachining and Microfabrication.

[7] Tohmori Y., Yoshikuni Y., Ishii H., Kano F., Tamamura T., Kondo Y., Yamamoto M. (1993). Broad-range wavelength-tunable superstructure grating (SSG) DBR lasers. IEEE J. Quantum Electron..

[8] Jayaraman V., Chuang Z.M., Coldren L.A. (1993). Theory, design, and performance of extended tuning range semiconductor lasers with sampled gratings. IEEE J. Quantum Electron..

[9] Mason B., Fish G.A., DenBaars S.P., Coldren L.A. (1999). Widely tunable sampled grating DBR laser with integrated electroabsorption modulator. IEEE Photonics Technol. Lett..

[10] Senturia S.D., Day D.R., Butler M.A., Smith M.C. (2005). Programmable diffraction gratings and their uses in displays, spectroscopy, and communications. J. Microlithogr. Microfabr. Microsyst..

[11] Schueller O.J.A., Duffy D.C., Rogers J.A., Brittain S.T., Whitesides G.M. (1999). Reconfigurable diffraction gratings based on elastomeric microfluidic devices. Sens. Actuators A Phys..

[12] Wong C.W., Jeon Y., Barbastathis G., Kim S.G. (2004). Analog piezoelectric-driven tunable gratings with nanometer resolution. J. Microelectromech. Syst..

[13] Shih W.C., Kim S.G., Barbastathis G. (2006). High-resolution electrostatic analog tunable grating with a single-mask fabrication process. J. Microelectromech. Syst..

[14] Yu Y.T., Yuan W.Z., Li T.P., Yan B. (2010). Development of a micromechanical pitch-tunable grating with reflective/transmissive dual working modes. J. Micromech. Microeng..

[15] Tormen M., Peter Y.A., Niedermann P., Hoogerwerf A., Shea H., Stanley R. (2006). Deformable MEMS grating for wide tunability and high operating speed. MOEMS-MEMS 2006 Micro and Nanofabrication.

[16] Yang Y.S., Lin Y.H., Hu Y.C., Liu C.H. (2008). A large-displacement thermal actuator designed for MEMS pitch-tunable grating. J. Micromech. Microeng..

[17] Aschwanden M., Beck M., Stemmer A. (2007). Diffractive transmission grating tuned by dielectric elastomer actuator. IEEE Photonics Technol. Lett..

[18] Zhang W.M., Yan H., Peng Z.K., Meng G. (2014). Electrostatic pull-in instability in MEMS/NEMS: A review. Sens. Actuators A Phys..

[19] Shih W.C., Hidrovo C., Kim S.G., Barbastathis G. Optical diversity by nanoscale actuation. Proceedings of the 2003 Third IEEE Conference on Nanotechnology (IEEE-NANO 2003).

[20] Elad M., Feuer A. (1997). Restoration of a single superresolution image from several blurred, noisy, and undersampled measured images. IEEE Trans. Image Process..

[21] Goodman J.W., Gustafson S.C. (1996). Introduction to fourier optics. Opt. Eng..

[22] Wu J., Wang S., Miao J. (2008). A MEMS Device for Studying the Friction Behavior of Micromachined Sidewall Surfaces. J. Microelectromech. Syst..

[23] Wu J., Wang S., Miao J. (2009). Friction characteristics of the curved sidewall surfaces of a rotary MEMS device in oscillating motion. J. Micromech. Microeng..

[24] Bell D.J., Lu T., Fleck N.A., Spearing S.M. (2005). MEMS actuators and sensors: observations on their performance and selection for purpose. J. Micromech. Microeng..

[25] Legtenberg R., Groeneveld A., Elwenspoek M. (1996). Comb-drive actuators for large displacements. J. Micromech. Microeng..

[26] Yu Y., Yuan W., Sun R., Qiao D., Yan B. (2010). A strategy to efficiently extend the change rate of period for comb-drive micromechanical pitch-tunable gratings. J. Microelectromech. Syst..

[27] Gao J., Yeo L., Chan-Park M.B., Miao J., Yan Y., Sun J., Lam Y., Yue C. (2006). Antistick postpassivation of high-aspect ratio silicon molds fabricated by deep-reactive ion etching. J. Microelectromech. Syst..

[28] Fu L., Miao J., Li X., Lin R. (2001). Study of deep silicon etching for micro-gyroscope fabrication. Appl. Surface Sci..

[29] Iliescu C., Miao J. (2003). One-mask process for silicon accelerometers on Pyrex glass utilising notching effect in inductively coupled plasma DRIE. Electron. Lett..

[30] Chen B., Miao J. (2007). Influence of deep RIE tolerances on comb-drive actuator performance. J. Phys. D Appl. Phys..

[31] Wong C.W., Jeon Y., Barbastathis G., Kim S.G. (2003). Analog tunable gratings driven by thin-film piezoelectric microelectromechanical actuators. Appl. Opt..

[32] Yi S., Kim S., Song J. (2013). Analysis of the effect of space radiations on the nematode, *Caenorhabditis elegans*, through the simulated space radiation. Int. J. Astron. Astrophys..

[33] Shaw T., Corliss J., Gundo D., Mulenburg G., Breit G., Griffith J. Simulation of shuttle launch G forces and acoustic loads using the NASA Ames Research Center 20G centrifuge. Proceedings of the 18th Space Simulation Conference on Space Mission Success Through Testing.

[34] Shea H.R. (2006). Reliability of MEMS for space applications. MOEMS-MEMS 2006 Micro and Nanofabrication.

